# Superatoms as Superior Catalysts: ZrO versus Pd

**DOI:** 10.1002/smll.202409289

**Published:** 2025-01-05

**Authors:** Mehmet Emin Kilic, Puru Jena

**Affiliations:** ^1^ Department of Physics Virginia Commonwealth University Richmond VA 23284‐2000 USA

**Keywords:** single‐atom catalysts, single‐superatom catalysts, superatoms, ZrO

## Abstract

Single‐atom catalysts are the focus of studies for over a decade due to their enhanced reactivity at smaller sizes. However, they have limitations as they offer only one active site, which may not be sufficient for reactions requiring the co‐adsorption of multiple reactants. Additionally, atoms can migrate on a substrate and coalesce, resulting in decreased reactivity. Here, an alternate path, a single‐superatom catalyst is provided. Superatoms are clusters of atoms that mimic the chemistry of atoms even if they do not contain a single atom whose chemistry they mimic. Motivated by an experimental paper on the photoelectron‐spectroscopy of negatively charged ions where ZrO is found to mimic properties of a Pd atom, first the reaction of Pd and ZrO with small molecules in the gas‐phase is studied and found that ZrO not only mimics the chemistry of Pd, but is able to activate these molecules more strongly than Pd. A detailed first‐principles study of CO_2_ reduction (CO_2_‐RR) and hydrogen evolution reactions (HER) on Pd and ZrO supported on graphene, Au(111), and Cu(111) surfaces shows that superatoms are indeed superior catalysts. The ability to design numerous superatoms by varying size and composition offers a promising new paradigm for catalyst design and synthesis.

## Graphical abstract

ZrO, a superatomic cluster mimicking the chemistry of the Pd atom is shown to outperform its property in binding, activating, and CO_2_ conversion when supported on substrates like graphene, Au(111), and Cu(111) ‐ highlighting the potential of single superatom catalysts.

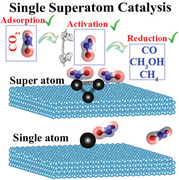

## 1 Introduction

There is considerable interest in developing efficient and inexpensive catalysts made of earth‐abundant elements as ≈90% of all commercially produced chemical products require some form of catalysts. Currently, these are made of expensive elements such as Pd, Pt, and Rh. In particular, developing inexpensive catalysts to convert CO_2_ to value‐added products is of great importance as it is a greenhouse gas and a primary contributor to global warming.^[^ ^1^ ^]^ As reactive sites in a catalyst are low‐coordinated atoms, supported metal nanoparticles have been the widely used type of heterogeneous catalysts in industrial processes.^[^ ^2^, ^3^ ^]^ However, the nanoparticles do not have uniform size, and the size variation not only reduces the metal atom efficiency but also frequently leads to undesired side reactions. The ultimate size of a nanoparticle is a single atom.^[^ ^4^ ^]^ More than a decade ago, the focus changed to single‐atom catalysts where the metal atoms are singly dispersed on a substrate.^[^ ^5^ ^]^ They not only increase reactivity but also minimize the number of inactive atoms in a nanoparticle while simultaneously reducing unwanted side reactions. Single‐atom catalysts (SACs), however, have limitations; they only offer a single active site that may not be sufficient for reactions requiring the co‐adsorption of multiple reactants. So, the challenge has been to find inexpensive catalysts, made of earth‐abundant elements, that have the desired property of a SAC, but offer multiple active sites. With lessons learned from cluster science, this work offers such an alternative. Extensive research on atomic clusters in the past decades has demonstrated that their properties can be tailored, one atom at a time. With specific size and composition, atomic clusters can be created to mimic the properties of atoms. Such clusters, termed as superatoms, not only outperform the reactivity of the atoms they mimic but also can serve as building blocks of novel materials.^[^ ^6^, ^7^, ^8^, ^9^, ^10^ ^]^ Note that while all superatoms are clusters, not all clusters qualify as superatoms. We propose a paradigm shift in catalyst design and synthesis by showing that a single superatom can outperform a single atom in converting CO_2_.

In a seminal paper published in 2010 in the Proceedings of the National Academy of Sciences, Castleman, and co‐workers showed that the photoelectron spectroscopy of negatively charged Pd ion is in agreement with its isoelectronic molecular counterpart ZrO.^[^ ^11^ ^]^ Note that atomic configurations of Pd, Zr, and O are[Kr]4d^10^,[Kr]4d^2^ 5s^2^, and [He]2s^2^ 2p^4^, respectively. Thus, ZrO has the same number of electrons in its outermost orbits as does a single Pd atom, namely 10. Based on this analogy, the authors suggested that ZrO can be termed as a superatom and serve as a replacement catalyst for Pd, although the authors did not study any chemical reaction to support this suggestion. In Table S1 (Supporting Information), we compare some of the electronic properties [e.g., electron affinity (EA), ionization potential (IP), energy levels of the highest occupied molecular orbital (HOMO) and the lowest unoccupied molecular orbital (LUMO), and the energy gap between HOMO and LUMO] of Pd, Zr, O atoms and ZrO dimer. Tyo et al. studied the reactivity of Pd^+^ and ZrO^+^ with small hydrocarbons.^[^ ^12^ ^]^ They found ZrO^+^ to behave similarly to that of Pd^+^ in reactions with ethane and propane, primarily through C─C bond scission and hydrogen abstraction, suggesting that ZrO^+^ could serve as a cost‐effective alternative to Pd. Later, Behera et al.^[^ ^13^ ^]^ showed that the reactivity of Pd*_n_* clusters (*n *= 1–5) with H_2_, O_2_, and CO is different from that of (ZrO)*_n_* clusters. A recent work by Ye et al.^[^ ^14^ ^]^ showed that (ZrO)*_n_* (*n *= 2–4) clusters did not improve the catalytic performance for CO oxidation. Several papers have since been published that demonstrate the catalytic potential of isolated clusters.^[^ ^15^, ^16^, ^17^, ^18^, ^19^, ^20^, ^21^, ^22^, ^23^, ^24^, ^25^, ^26^, ^27^ ^]^ However, all these studies are on clusters in the gas phase.

For practical applications, clusters need to be supported on a substrate. Whether superatomic clusters can serve as practical catalysts raises some fundamental questions: First, would the superatom retain its structure and properties, once supported on a substrate? Second, would the reactions of a superatom with molecules be the same as its corresponding isoelectronic atom, while both are supported on the same substrate? Third, would the results be sensitive to the substrate? We have recently shown that Li_3_O, a superalkali mimicking the chemistry of the alkali atom Li, not only retains its geometry when supported on a substrate but also binds and activates a CO_2_ molecule more strongly than the Li atom.^[^ ^28^ ^]^ Note that neither the Li atom nor its Li_3_O superatom are traditional catalysts. To our knowledge, no studies, either theoretical or experimental, have been carried out to see if the superatomic ZrO would retain its structure when deposited on a substrate; whether the results are substrate‐dependent; and most importantly, is the reaction of this superatom analogous to that of its isoelectronic atom, Pd?

We carried out such a study by adsorbing Pd and ZrO on three different substrates [e.g., graphene (Gr), Au(111), and Cu(111)] and reacting them with CO_2_. Note that a single‐superatom catalyst should satisfy three important steps in a catalytic process: the catalysts i) should be strongly bound to the substrate, ii) be able to bind the molecules with energies intermediate between physisorption and chemisorption while simultaneously activating them, and iii) show superior catalytic performance with low overpotentials. Herein, we present some very encouraging results. We show that i) isolated ZrO can activate small molecules such as H_2_, O_2_, N_2_, NO, CO, and CO_2_ more strongly than its isoelectronic atom, Pd; ii) ZrO is bound to the substrate more strongly than Pd. iii) ZrO not only retains its structure when deposited on substrates but also outperforms its isoelectronic atom, Pd in activating CO_2_; iv) ZrO shows better catalytic performance with very low overpotential compared to its atomic counterpart, Pd; and v) the results are not very sensitive to the substrates.

In the following, we outline our theoretical methods and study the interaction of small molecules with isolated and supported atom, Pd, and its corresponding superatom, ZrO. The encouraging results open the door for exploring single‐superatom catalysis as an alternate to single‐atom catalysis.

## 2 Results and Discussion

Superatom should mirror the chemistry of the atoms it represents. A key question that arises is what defines this “chemistry”? Although it may be difficult to account for all the properties simultaneously, we can focus on key characteristics, such as valence electron count, the energy levels of the HOMO and LUMO, EA, IP, and orbital shapes. We first compare these properties for an isolated Pd atom and its superatom ZrO in **Table** **1** using different basis sets and in Figure S2 (Supporting Information). Note that, in addition to having the same number of valence electrons (10), Pd and ZrO exhibit comparable HOMO, LUMO, EA, and IP values. However, their orbital shapes differ, which are influenced by ZrO being di‐atomic and polar as well as different spin multiplicities, with Pd and ZrO having magnetic moments of 0 and 2 *μ_B_*, respectively. To see if these features allow ZrO to mimic the chemistry of Pd, we further study their reaction with small molecules in the gas phase.

**Table 1:** Highest occupied molecular orbital (HOMO), lowest unoccupied molecular orbital (LUMO), electron affinity (EA), ionization potential (IP), and total number of valence electrons (VE) of Pd and its superatom counterpart, ZrO using various basis sets, namely Def2tzvp, SDD, and LanL2tz for Pd and Zr transition metals, and Def2tzvp for the oxygen atom.

	Pd			ZrO		
	Def2tzvp	SDD	LanL2tz	Def2tzvp	SDD	LanL2tz
HOMO [eV]	−4.93	−5.25	−4.74	−4.64	−4.67	−4.72
LUMO [eV]	−2.53	−2.74	−2.38	−2.84	−2.85	−2.81
EA [eV]	0.41	0.77	0.21	1.21	1.28	1.16
IP [eV]	8.57	8.69	8.58	7.33	7.40	7.41
VE	10 (Pd: 4d^10^)			10 (Zr:4d^2^ 5s^2^, O: 2s^2^ 2p^4^)		

Next, we study if Pd and ZrO possess similar properties when supported on different substrates (Gr, Au(111) and Cu(111)). Finally, we study the catalytic properties of supported Pd and ZrO by focusing on CO_2_‐RR and HER.

### 2.1 Interaction of Isolated Pd Atom and Its Superatom, ZrO, with H_2_, O_2_, N_2_, NO, CO, and CO_2_

**Figure 1:**
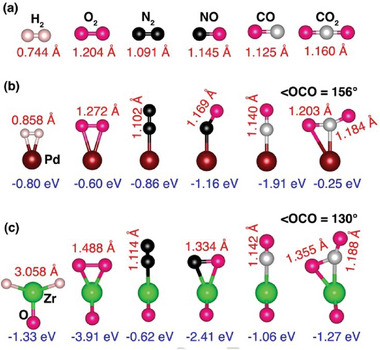
The equilibrium atomic configurations of a) H_2_, O_2_, N_2_, NO, CO, and CO_2_ in the gas phase, b) interacting with an isolated Pd atom, and c) ZrO superatom. The calculated bond lengths and binding energies are presented.

In **Figure** **1** we provide the equilibrium bond lengths of free H_2_, O_2_, N_2_, NO, CO, and CO_2_ molecules and show how they change including the ∠OCO bond angle from their pristine values when interacting with isolated Pd and ZrO. We note that bond distortions in these small molecules can be considered as descriptors for their activation. The results show that all the bond lengths are elongated when interacting with Pd and ZrO. Importantly, the ZrO superatom activates the molecules stronger than the atoms as seen from greater changes in the bond lengths. For instance, the O─O bond length, initially at 1.204 Å in O_2_ molecule, extends to 1.272 and 1.488 Å when bound to Pd and ZrO, respectively. Likewise, the C─O bond length of 1.160 Å in CO_2_ molecule extends to 1.203 and 1.355 Å when bound to Pd and ZrO, respectively. Moreover, the ∠OCO bond angle, which is initially 180°, reduces to 156° and 130° when bound to Pd and ZrO, respectively.

Also given in this figure are the binding energies of the molecules with the reacting species. This is defined as *E*_b _= *E*(*MX*) − *E*(*M*) – *E*(*X*), *M *= Pd, ZrO and *X *= H_2_, O_2_, N_2_, NO, CO, and CO_2_. Here, negative binding energy indicates that *X* is bound to *M*. In all cases, the binding energy is larger when molecules interact with the ZrO superatom compared to the Pd atom. Thus, although Pd and ZrO have similar chemistry, they exhibit different behaviors when interacting with gas molecules. In fact, in the gas phase, the superatom outperforms the atom in activating the molecules.

### 2.2 Structure and Properties of Pd and ZrO Supported on Graphene, Au(111), and Cu(111) Surfaces

**Figure 2:**
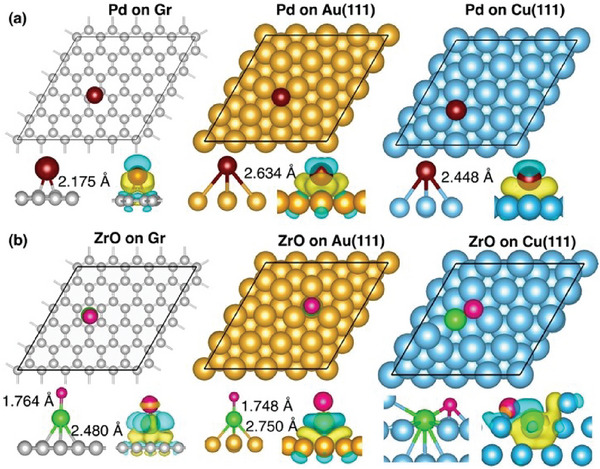
The equilibrium atomic configurations of a) Pd and b) ZrO supported on graphene, Au, and Cu surfaces. The lower panel of each figure illustrates the charge density differences, with yellow regions indicating charge accumulation and sky‐blue regions indicating charge depletion.

As mentioned earlier, catalysts whether they are a single atom or a single superatom, need to be supported on substrates. The interaction of the substrate with the supported atoms/superatoms is expected to change their properties. Thus, the interaction of the supported species with molecules is expected to be different from that in the gas phase. We first study the site occupancy and properties of Pd and its superatom ZrO supported on different substrates and see if these species are adsorbed on the surface or embedded into the host by replacing the host atoms. To determine the most stable adsorption site, we placed Pd and ZrO in various positions, including hollow site, on‐top site, and bridge site. The equilibrium configurations are presented in **Figure** **2**. The most stable position for a single Pd atom on the graphene surface is the bridge site above the bond between two carbon atoms, with a binding energy of −1.29 eV, which is agreement with the previous result.^[^ ^29^ ^]^ ZrO, on the other hand, prefers the hollow site, with a slightly larger binding energy of −1.47 eV. Here, the Zr atom is bound to the surface with the Zr─O bond perpendicular to the surface. On the Au(111) surface, both Pd and ZrO show a strong preference for adsorption on the hollow‐fcc site with a binding energy of −3.17 and −4.53 eV, respectively. Similar to the graphene surface, the Zr─O bond is oriented vertically when attached to Au(111), bringing Zr in close proximity to the surface atoms. On the Cu(111) surface, the Pd atom prefers to bind on the hollow‐fcc site with a binding energy of −3.67 eV while a ZrO super‐atom prefers the subsurface layer rather than the surface layer with a binding energy of −4.36 eV. The higher binding energies of the superatom ensure its enhanced structural stability on the surfaces.

We further carried out a Bader charge analysis to determine the charge transfer when Pd (ZrO) is deposited on the surfaces. The results, provided in **Table** **2**, show that consistent with the larger binding energy of the superatom compared to the atom, the charge transfers between the ZrO superatom and the surfaces are higher compared to that between the Pd atom and the surface. This is also confirmed by the analysis of charge density differences (Figure 2, lower panels). The larger electron transfer from the superatom locally increases the electron density on the surfaces, which may facilitate their catalytic activity. Hence, despite the similarity in the chemistry of Pd and ZrO, their interaction with the surfaces is distinctly different. For instance, the ZrO superatom forms stronger bonds with the surfaces, facilitating higher charge transfer compared to the atom.

**Table 2:** Calculated binding energies in eV and charge transfers in electrons when Pd (ZrO) is bound to graphene (Gr), Au(111), and Cu(111) surfaces. The positive/negative sign of the charge transfer indicates that the atom (superatom) gains/loses electrons from/to the surfaces.

	*E*_B_ [eV]			Δ*q* [e]		
	Gr	Au	Cu	Gr	Au	Cu
Pd ZrO	−1.29 −1.47	−3.17 −4.53	−3.67 −4.36	0.18 0.89	−0.06 −1.07	+0.21 −0.65

### 2.3 Single‐Atom (Pd) versus Single‐Superatom (ZrO) Catalysts

Our focus here is on CO_2_ reduction reactions (CO_2_‐RR). In addition to the importance of addressing global warming, the study of CO_2_‐RR is interesting for the following two reasons: i) CO_2_ is highly stable due to its linear molecular structure and the strong double bonds between the carbon and the oxygen atoms, necessitating significant energy for bond breaking and chemical reactions with other substances. ii) CO_2_‐RR is generally considered more complex than the oxygen evolution reaction (OER), the oxygen reduction reaction (ORR), or the hydrogen evolution reaction (HER). It involves multiple electron and proton transfers, resulting in a variety of value‐added products such as CO, methane, methanol, and others, each requiring different numbers of electrons and protons. We study the adsorption, activation, and reaction pathways of CO_2_ molecules on Pd and ZrO‐doped surfaces.

#### 2.3.1 A Free CO_2_ and CO_2_^−^ Molecule

**Figure 3:**
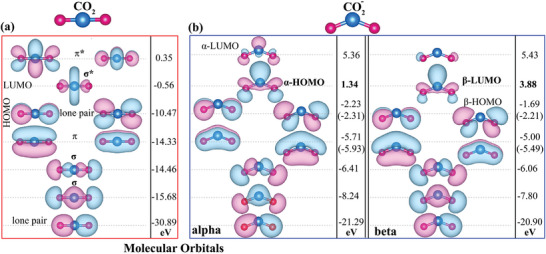
Molecular orbitals (MO) of a) CO_2_ and b) CO_2_^‐^ molecules.

To understand the adsorption and activation of a CO_2_ molecule, we first study how its bond length, bond angle, and molecular orbital structure change when charge transfer occurs. We performed the spin‐polarized DFT calculations for a free CO_2_ molecule and its mono‐anion (CO_2_^−^). The results presented in **Figure** **3** indicate that the free CO_2_ molecule has a linear arrangement with a *π*‐type electron configuration that is delocalized. In terms of its molecular orbitals, the HOMO of CO_2_ is characterized by non‐bonding properties involving 2p atomic orbitals around the oxygen atoms, while HOMO‐1 exhibits a delocalized *π*‐type bonding orbital. On the other hand, the LUMO is a *σ*‐type anti‐bonding orbital primarily influenced by the carbon atom, and LUMO+1 involves *π*‐type anti‐bonding orbitals across three atoms. Thus, the activation of the CO_2_ molecule can be described in two ways: i) CO_2_ undergoes activation by introducing electrons into its LUMO, ii) The lone pair electrons from the HOMO of the CO_2_ molecule are transferred to the active site. The lone pair electrons, with an energy level of −10.47 eV, would face a considerable barrier in transferring electrons from this deep energy level. However, the energy levels of σ* (−0.56 eV) and π* (0.35 eV) imply that, in the majority of scenarios, activation predominantly occurs via the first type. Upon adding an extra electron, the CO_2_ molecule bends, resulting in an O─C─O angle of 137°. This significantly reduces the energy of the 2p orbital, enabling it to accept electrons from the surface of the HOMO.

#### 2.3.2 Adsorption of CO_2_

**Figure 4:**
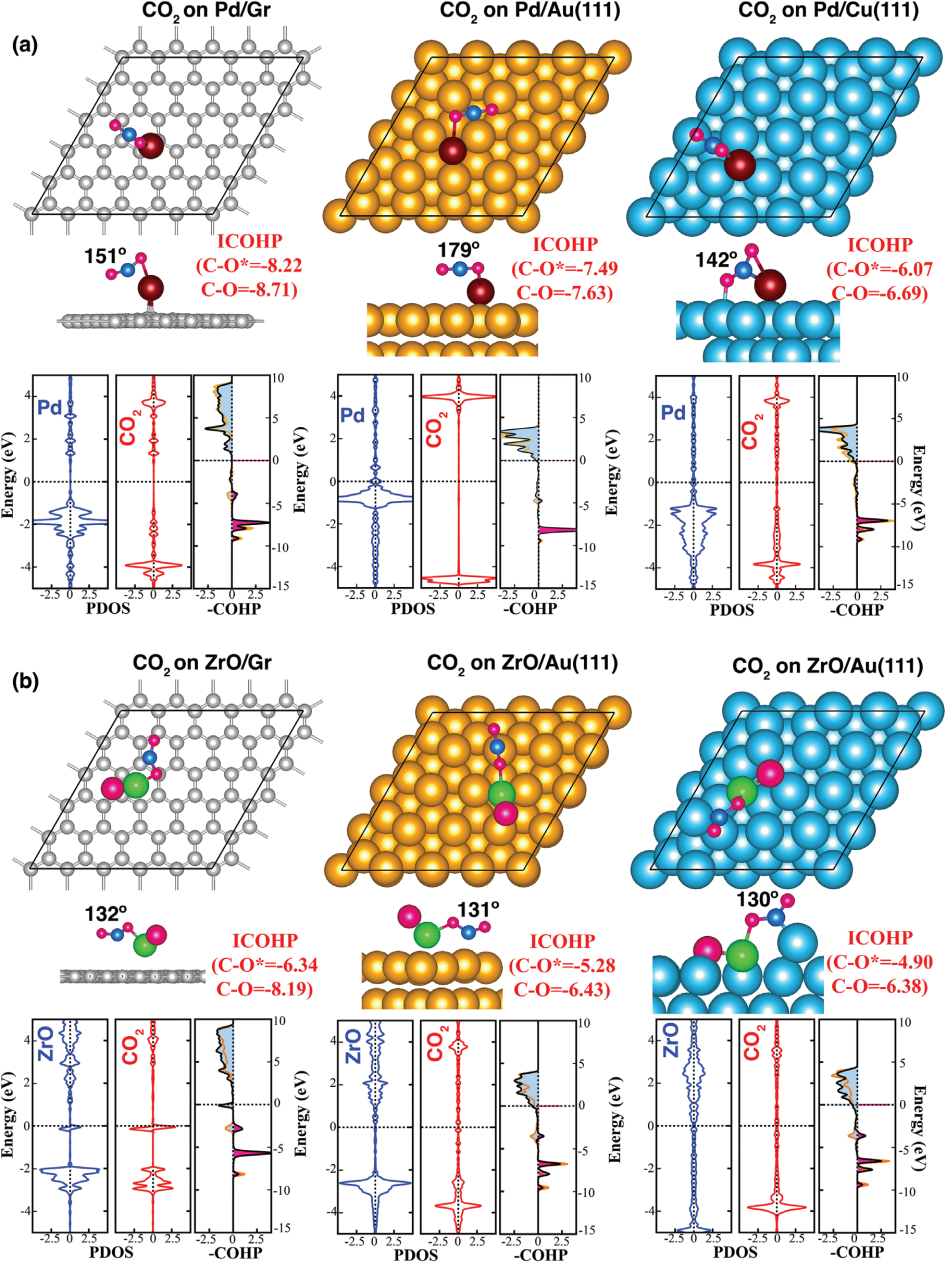
The equilibrium atomic configurations with top and side views, PDOS, and COHP results of CO_2_ adsorbed on a) Pd single atom and b) ZrO superatom supported on graphene, Au(111), and Cu(111) surfaces. The PDOS of the Pd (ZrO) and CO_2_ molecule on CO_2_ adsorbed Pd (ZrO)Gr, Au, and Cu are depicted in blue and red colors, respectively. For bonding and antibonding interactions, the COHP of the C─O bonds in the adsorbed CO_2_ molecule is presented in plots using black and orange colors. Bonding interactions, represented by positive ─COHP values, are shaded in pink, whereas antibonding interactions, indicated by negative ‐COHP values, are shaded in sky‐blue. The ICHOP values for C─O* (O* denoting the oxygen atom bonded to the Pd (Zr) atom) and C─O bonds are depicted.

A critical step to determine the catalytic mechanism is to ensure that the molecules are simultaneously adsorbed on and activated by the catalysts. This is crucial for subsequent reaction pathways. We investigated CO_2_ adsorption on Pd and ZrO supported on graphene, Au, and Cu surfaces. Several CO_2_ adsorption configurations on selected sites of the surfaces as well as the approach of the ZrO are considered. The most stable CO_2_ configuration on the Pd and ZrO doped surfaces is presented in **Figure** **4**. Both the Pd atom and ZrO superatom are found to be the active sites for CO_2_ binding and activation, rather than the surface atoms of the substrates. The binding energies of CO_2_ molecule on Pd (ZrO) doped graphene, Au, Cu surfaces are −0.68 (−1.47), −0.34 (−0.83), −0.33 (−0.81) eV, respectively. These results already prove that a superatom outperforms the atom whose chemistry it mimics, thus, validating the first step of a catalytic process, that is, binding to the molecules.

#### 2.3.3 Activation of CO_2_

The next step is the activation of the CO_2_ molecule where the symmetry is indicative of its activation. This requires not only the stretching of the C─O bond but also the bending of the ∠OCO angle from its normal 180°. The calculated C─O/C─O* (where O* refers the oxygen atom bound to Pd or Zr on the surfaces) bond lengths of the adsorbed CO_2_ molecule on Pd (ZrO) doped graphene, Au, Cu surfaces are 1.199/1.241 Å (1.215/1.329 Å), 1.174/1.183 Å (1.207/1.293 Å), 1.229/1.236 Å (1.217/1.312 Å), respectively. This indicates that upon adsorption of the CO_2_ molecule, one of the C═O bonds becomes more elongated (or weakened), thereby breaking its initial symmetry. Accordingly, the corresponding ∠OCO bond angles are 151° (132°), 179° (131°), 142° (130°) (Table 3). A larger deviation from the symmetric bond lengths and angles of a free CO_2_ molecule are observed when adsorbed on ZrO‐supported surfaces compared to Pd‐supported surfaces. Thus, asymmetry or altered bond lengths would further make CO_2_ more prone to chemical reactions.

To further obtain an in‐depth understanding of the activation of the CO_2_ molecule on the corresponding surface, the crystal orbital Hamilton population (COHP) and the integral value of COHP below the Fermi level (ICOHP) analyses were performed. The bonding and antibonding states are represented by positive and negative values in ‐COHP, respectively. As ICOHP is an index that reflects the interaction strength between two atoms, we calculated ICOHP for the C─O bonds in the adsorbed CO_2_ molecule. The ‐COHP and corresponding ICOHP values are given in Figure 4. The calculated ICOHP values for the C─O*/C─O bonds in the CO_2_ molecule adsorbed on Pd (ZrO) supported graphene, Au, and Cu are as follows: −8.22/−8.71 (−6.34/−8.19), −7.49/−7.63 (−5.28/−6.43), −6.07/−6.69 (−4.90, −6.38), respectively. These values are lower compared to the CO bonds in a free CO_2_ molecule (ICOHP^C─O ^_=_ −9.16). Moreover, the ICOHP values for the bonds of Pd─O* (Zr─O*) in the CO_2_ molecule adsorbed on Pd (ZrO) supported graphene, Au, and Cu are −0.49 (−1.78), −0.17 (−1.47), and −0.20 (−1.79), respectively. These findings suggest several key points: i) The strength of CO bonds decreases when a CO_2_ molecule is adsorbed onto the surfaces compared to free CO_2_, ii) The strength of C─O* bonds is lower than that of C─O, iii) Both C─O* and C─O bond strengths are lower when CO_2_ molecule is adsorbed on ZrO supported surfaces compared to that on Pd supported surfaces. iv) The results for ICOHP^Zr─O*^ and ICOHP^Pd─O*^ indicate that the CO_2_ molecule is more strongly bound to ZrO than to Pd. Therefore, ZrO again outperforms the Pd atom in activating the CO_2_ molecule on all three surfaces.

We further analyzed the density‐of‐state (DOS) and partial density‐of‐state (PDOS) for CO_2_ adsorbed on Pd and ZrO‐supported graphene, Au, and Cu surfaces. The results are presented in Figures 4 and S3 (Supporting Information). New hybridized energy levels near the Fermi level, namely, d–π* (occ.) and d–π* (unocc.) formed by the interaction between π antibonding orbitals of CO_2_ and *d* orbital of Pd or Zr atom. This interaction will help strengthen CO_2_ adsorption and reduce the total energy of the overall system. One can easily see a large overlapping area for d–π* when CO_2_ adsorbed on ZrO‐supported surfaces, implying a strong interaction between CO_2_ and ZrO atoms on the surfaces (**Table** **3**).

**Table 3:** Calculated CO_2_ binding energies (*E*_B_) in eV, charge transfers (Δ*q*) in electrons, ∠OCO bond angles in degrees, and ICOHP of C─O* (* denoted the oxygen atom bound to Pd or Zr) bond in CO_2_ molecule when CO_2_ binds to the Pd atom and ZrO superatom supported on graphene (Gr), Au(111), and Cu(111) surfaces. The positive sign in the charge transfer indicates that the CO_2_ molecule gains electrons from the surfaces.

		Substrate		
		Gr	Au	Cu
*E_B_*	Pd	−0.68	−0.34	−0.33
[eV]	ZrO	−1.47	−0.83	−0.81
Δ*q*	Pd	+0.29	−0.02	+0.40
[e]	ZrO	−0.93	−0.56	+0.73
∠OCO	Pd	151	179	142
[°]	ZrO	132	131	130
ICOHP	Pd	−8.22	−7.49	−6.07
	ZrO	−6.34	−5.28	−4.90

#### 2.3.4 CO_2_‐RR and Reaction Pathways

**Figure 5:**
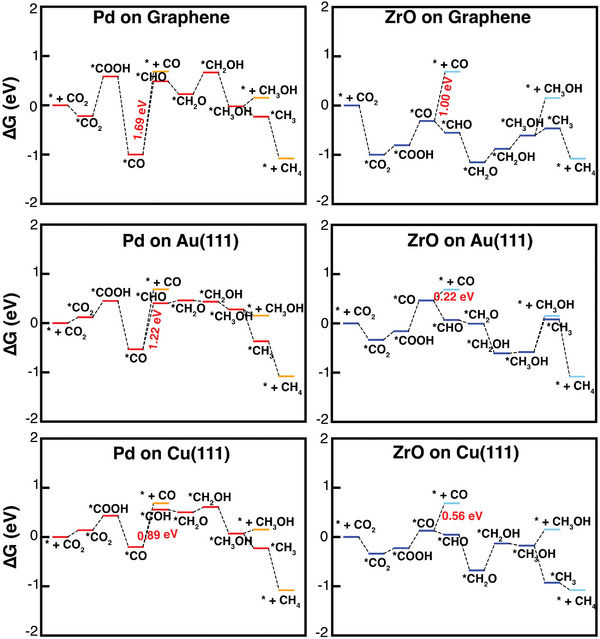
Calculated Gibbs free energy change (Δ*G*) diagrams of the electrochemical CO_2_‐RR on the Pd (ZrO) doped graphene, Au, and Cu surfaces at zero potential.

**Figure 6:**
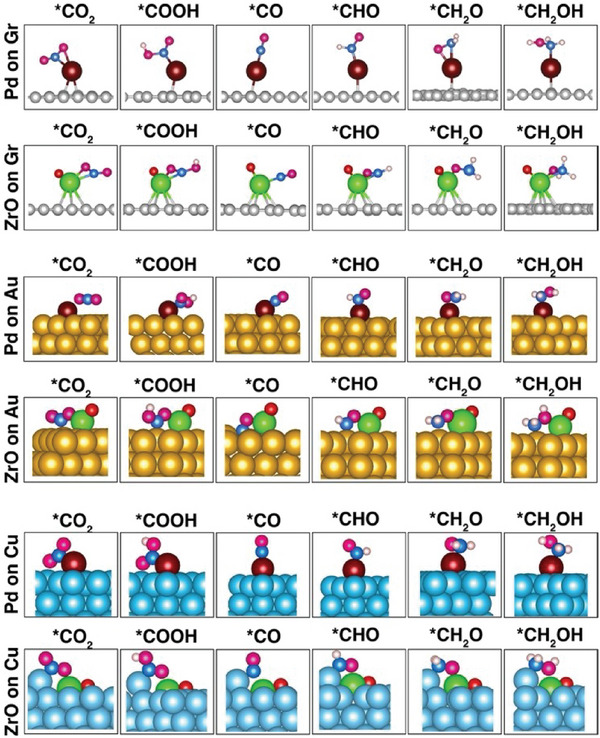
Equilibrium atomic configuration of *CO_2_, *COOH, *CO, *CHO, *CH_2_O, and *CH_2_OH intermediates on the Pd and ZrO doped Gr, Au, and Cu surfaces.

In studying the CO_2_‐RR to identify potential products, we calculated Gibbs free energy changes. This approach provides an understanding of the thermodynamic feasibility of various product formations during CO_2_‐RR. This starts with the hydrogenation catalysis of CO_2_ molecules through multiple proton and electron transfer steps involving many possible intermediates. Taking into account the zero‐point energy and vibration entropy, we have drawn the computed free energy diagrams of the electrochemical CO_2_ reduction to C_1_ products (CO, CH_3_OH, and CH_4_) at zero potential in **Figure** **5**. The geometrical structures of key reaction intermediates in the electrochemical CO_2_‐RR process are presented in **Figure** **6**. All energies including the total energies, zero‐point energies, and Gibbs free energies of the key reaction intermediates are given in Tables S2,S3 (Supporting Information).

In the following, we discuss the free energy changes (Δ*G* = *G*(*CO_2_) – *G*(* + CO_2_)) relative to the * + CO_2_ → *CO_2_ reaction; the binding energy in this process has been given before. Here, * represents the clean surface (e.g., Pd or ZrO supported surface), *CO_2_ represents the CO_2_ molecule adsorbed on the substrate, and CO_2_ refers to the molecule in the gas phase. The free energy changes in Pd (ZrO) doped graphene, Au, Cu substrates are −0.22 (−1.00), 0.12 (−0.33), and 0.14 (−0.34) eV, respectively. We note that the negative energies correspond to spontaneous activation and formation of *CO_2_ from its gas phase on the surfaces.

The first hydrogenation step in the electro‐reduction of CO_2_ involves binding with a proton and gaining one electron, resulting in the formation of two potential intermediates according to the binding position of a proton: one where a hydrogen atom is bound to one of the oxygen atoms in *CO_2_, resulting in *COOH, and another where the hydrogen atom is bound to the carbon atom, resulting in *CHOO. For all the studied surfaces, the formation of the *COOH intermediate is favorable. The free energy required for the formation of the *COOH from the reference state (* + CO_2_) on the Pd (ZrO) supported on graphene, Au, and Cu surfaces are calculated to be 0.58 (−0.81), 0.45 (−0.16), and 0.43 (−0.23) eV, respectively. The higher negative Δ*G* corresponds to a more favorable formation of intermediate species on the corresponding surfaces, and thereby, the formation of the *COOH intermediate is spontaneous on the ZrO‐doped surfaces.

The second hydrogenation step following the formation of the *COOH, can produce the 2e^−^ CO product through the following reaction pathway *COOH + H^+^ + e^−^ → *CO + H_2_O → * + CO. The free energy changes for the reaction of *COOH + H^+^ + e^−^ → *CO + H_2_O, are calculated to be −1.59 (0.49), −0.98 (0.62), −0.64 (0.35) eV for the Pd (ZrO) supported on graphene, Au, and Cu surfaces, respectively. This indicates that the free energy required for the formation of *CO on the Pd‐doped surfaces is highly negative, whereas on the ZrO‐doped surfaces, the free energy is relatively small and positive. Importantly, a highly negative free energy change for adsorption indicates that the process is thermodynamically very favorable and occurs spontaneously on the surface. However, this also means that desorption would require a large positive free energy, making it unfavorable and energy‐intensive. Thus, it is desirable to have a negative free energy for CO adsorption as well as easy desorption from the surface. We also calculated the free energy changes for the reaction of *CO → * + CO which are 1.69 (1.00), 1.22 (0.22), 0.89 (0.56) eV on the Pd (ZrO) doped graphene, Au, Cu surfaces, respectively. The preferred energy for the desorption of *CO should be less than 1 eV. Thus, the ZrO‐doped surfaces, especially Au, are desirable for CO_2_‐RR to yield CO products.

In general, *CO is the important key intermediate for further reduction, and the selectivity is determined by the binding energy of *CO. The calculated CO binding energies on the Pd (ZrO) doped graphene, Au, and Cu surfaces are −2.19 (−1.50), −1.71 (−0.79), −1.48 (−1.12) eV, respectively. This indicates that CO binds more strongly to the Pd‐doped surfaces compared to the ZrO‐doped surfaces. If the catalyst binds *CO strongly, the catalytic active sites are poisoned where HER becomes dominant.

Instead of the desorption of *CO from the surfaces, further hydrogenation could lead to the formation of *CHO or *COH intermediates. The Pd (ZrO) doped surfaces primarily lead to the formation of *CHO intermediate rather than *COH. For the reaction of *CO + H^+^ + e^−^ → *COH, the calculated free energy changes on the Pd (ZrO) doped graphene, Au and Cu surfaces are 1.49 (−0.24), 0.94 (−0.40), 0.76 (−0.08) eV, respectively. The large positive free energy change on the Pd‐doped surfaces indicates that further hydrogenation is also feasible. In contrast, the negative free energy changes on the ZrO‐doped surfaces make them favorable for forming other potential products.

As a result, from a thermodynamic point of view, the path with the following intermediates *COOH→ *CO → *COH → *CH_2_O → *CH_2_OH is found to be the most preferable path. For the ZrO doped graphene, the potential determining step (PDS), that is, the step with the largest positive free energy change, is the step *COOH + H^+^ + e^−^→ *CO + H_2_O where the limiting potential is ‐0.49 eV and the overpotential is 0.62 eV. For the ZrO doped Au surface, the PDS is *CH_3_OH + H^+^ + e^−^ → *CH_3_ + H_2_O with a limiting potential of −0.66 eV and an overpotential of 0.79 eV. In the case of the ZrO doped Cu surface, the step is *CH_2_O + H^+^ + e^−^ → *CH_2_OH where the limiting potential is calculated as −0.63 eV and the overpotential is 0.76 eV. The ZrO superatom exhibits a low overpotential of less than 1 eV, making it a promising catalyst compared its single‐atom counterpart, Pd.

As the CO_2_‐RR involves various protonation steps, we also analyzed the formation of H* (* + H^+^ + e^−^ → *H), known as HER, which is a key intermediate. The free energy required for HER on the Pd (ZrO) doped graphene, Au, and Cu are 0.44 (−0.35), −0.03 (−0.31), −0.22 (−0.18) eV, respectively. The energies of the step close to zero are favorable for HER, indicating that ZrO outperforms Pd in HER when supported on graphene and Cu surfaces. As the CO₂‐RR competes with the HER, suppressing HER is a critical challenge in developing efficient CO₂‐RR processes. Because Δ*G* for the formation of *COOH is more negative compared to *H, it indicates that the formation of the *COOH intermediate is more favorable on the superatom doped surfaces.

## 3 Conclusion

A comprehensive theoretical study of the interaction of isolated and supported Pd and its superatomic counterpart ZrO on three different substrates—graphene, Au(111), and Cu(111)—using DFT yields the following results: i) Isolated Pd atoms and its isoelectronic ZrO superatom react similarly with small molecules such as H_2_, O_2_, N_2_, NO, CO, and CO_2_ suggesting that the atom and its corresponding superatom have similar chemistry. Furthermore, the ZrO superatom binds and activates the molecules more strongly than the Pd atom whose chemistry it mimics. Thus, the ZrO superatom can not only do what its corresponding Pd atom can but does it *better*. ii) When deposited on different substrates such as graphene, Au, and Cu, the ZrO superatom not only binds more strongly to the surface than the Pd atom but also remains intact. iii) As the conversion of CO_2_ to value‐added products is important for technology, the catalytic performance of Pd and ZrO supported on these substrates is studied by focusing on CO_2_‐RR. The results of the electrochemical CO_2_‐RR process on the Pd and ZrO doped surfaces show that the ZrO superatom exhibits higher activity and selectivity in CO_2_‐RR.

These results suggest a paradigm shift in catalyst design–from single‐atom catalysts to single–superatom catalysts. The advantages of a single‐superatom versus a single‐atom catalyst are several: i) The former offers multiple active sites for chemical reactions. ii) A superatom can be made of earth‐abundant elements and can replace rare, toxic, and expensive elements used as catalysts. iii) Even more important, the energetics of reaction pathways for CO_2_‐RR studied here for superatom ZrO are found to be more favorable than that of the Pd atom whose chemistry it mimics.

It should be pointed out that surface defects may also play a role in single‐superatom catalysis as superatoms can be trapped by defects as atoms are and the catalytic property of a trapped superatom may differ from those bound to a clean surface. This aspect will be investigated in a future work. Note, that ZrO may not be the only superatom that mimics the chemistry of Pd. It is likely that many other clusters can be designed to have similar properties. Here, machine learning approaches may be very useful. In summary, a single‐superatom can be a better catalyst than a single‐atom, opening the door to a new class of superior catalysts. It is hoped that this work will motivate further experimental and theoretical work in catalyst design and in the study of other processes not considered here.

## 4 Experimental Section

All calculations were carried out using spin‐polarized density functional theory (DFT). The interaction of isolated Pd atom and ZrO superatom with small molecules was studied using Gaussian‐16 code_._^[^ ^30^ ^]^ Effective core potentials for Pd and Zr atoms utilized the SDD and LanL2tz basis sets, while the def2‐tzvp basis set was used for all other atoms. The exchange‐correlation potential was accounted for by using the hybrid B3LYP functional.^[^ ^31^, ^32^, ^33^ ^]^ When investigating Pd and ZrO supported on graphene, Au(111), and Cu(111) substrates, the DFT‐based calculations were carried out using the Vienna Ab Initio Simulation Package.^[^ ^34^, ^35^ ^]^ Perdew–Burke–Enzerhof functional^[^ ^36^ ^]^ and the projector‐augmented wave^[^ ^37^ ^]^ were used to describe the exchange‐correlation potential and ion‐electron interactions, respectively. Long‐range dispersion force was accounted for using the DFT+D3 approach by Grimme.^[^ ^38^ ^]^ The energy and force tolerance were 10^−5^ eV and 0.01 eV Å^−1^, respectively. To study the site preference of Pd and ZrO on substrates as well as to examine the adsorption of CO_2_ molecules and the intermediates, supercells described in the Supporting Information (see Figure S1, Supporting Information) were created. The electronic structure was further studied by carrying out COHP analysis using the LOBSTER program^[^ ^39^, ^40^ ^]^ and the PDOS.

To determine the thermodynamical reaction energetics for the CO_2_ reduction reaction (CO_2_‐RR) along with the reaction pathways, the standard equilibrium potentials of electrochemical steps were calculated using a computational hydrogen electrode model.^[^ ^41^, ^42^ ^]^ The reference electrode in this study was the theoretical reversible hydrogen electrode (RHE) where at zero external potential versus RHE, protons and electrons were in equilibrium with H_2_ at 298.15 K at all pH values.

(1) $$H^{+}+e^{-}↔1/2\left(H_{2}\right)$$

In CHE, the chemical potential of the proton–electron pair *µ*(H^+^) + *µ*(e^−^) is equal to half of the chemical potential of gaseous hydrogen (1/2 *µ*(H_2_)) at *U* = 0 V. Thus, the chemical potential of the proton–electron pair could be calculated simply by computing the chemical potential of the gas phase H_2_.

To correct the electron energy, zero‐point energy (ZPE) and entropy (*S*) contribution were calculated. The free energy of a system is given by;

(2) $$\Delta G=\Delta E+\Delta E_{\mathrm{ZPE}}-T\Delta S+\Delta G_{U}+\Delta G_{\mathrm{pH}}$$

where Δ*E* represents the total energy change derived from the DFT calculations, Δ*E*_ZPE_ denotes the change in the zero‐point energy, *T* stands for the temperature (taken as room temperature, 298.15 K, in all the calculations), and Δ*S* indicates the change in entropy at temperature *T*. Δ*G*_U_ accounts for the applied electrode potential, and Δ*G*_pH_ reflects the effect of pH on Gibbs free energy, assuming a pH of zero under acidic conditions. Gibbs free energy corrections to gas‐phase molecules are obtained from the NIST database (https://cccbdb.nist.gov).

The overpotential (η) and limiting potential (*U*_L_) are important parameters for estimating the performance of a catalyst. The *U*_L_ is calculated using the equation: *U*_L = _−Δ*G*/e where −Δ*G* is the change in Gibbs free energy for the rate‐limiting step. The *η* for electrochemical CO_2_‐RR is calculated from *η* = *U*_eq_ − *U*_L_, where *U*_eq_ is the equilibrium potential (0.169 V for CH_4_).^[^ ^43^ ^]^

## Conflict of Interest

The authors declare no conflict of interest.

## Supporting information



Supporting Information

## Data Availability

The data that support the findings of this study are available from the corresponding author upon reasonable request.
