# Supplementary material for: Superatoms as Superior Catalysts: ZrO versus Pd
Source: Small. 2025 Jan 5;21(8):2409289. doi: 10.1002/smll.202409289 (PMC11855260; doi:10.1002/smll.202409289)
Supplement: Supplementary file 1 — Supporting Information [file SMLL-21-2409289-s001.pdf]

NANO · MICRO
small

Supporting Information

for *Small*, DOI 10.1002/smll.202409289

Superatoms as Superior Catalysts: ZrO versus Pd

*Mehmet Emin Kilic and Puru Jena**

Supporting Information:

Superatoms as Superior Catalysts: ZrO vs Pd

*Mehmet Emin Kilic and Puru Jena**

Physics Department, Virginia Commonwealth University, Richmond, VA 23284

Table S1 Electron affinity (EA), ionization potential (IP), highest occupied molecular orbital (HOMO) and lowest unoccupied molecular orbital (LUMO) energy levels, and the HOMO-LUMO energy gap (in eV) for single Zr, O, ZrO, and Pd calculated using B3LYP functional and Def2t basis set. The average values for Zr and O atoms are also presented.

	EA (eV)	IP (eV)	HOMO (eV)	LUMO (eV)	Egap (eV)
**Zr**	0.480	6.666	-4.610	-2.681	1.930
**O**	1.059	14.111	-9.165	-5.007	4.157
**ZrO (avg.)**	0.770	10.389	-6.887	-3.844	3.043
**ZrO**	0.697	8.087	-4.611	-2.826	1.785
**Pd**	0.408	8.574	-4.932	-2.533	2.398

Graphene (Gr), Au(111) and Cu(111) Slabs: To determine the preferred site of the Pd and ZrO on the graphene, Au(111), and Cu(111) substrates and the adsorption of the CO_2 molecules, it is essential to construct sufficiently large enough supercell where the spurious interactions between the images are small. In the case of graphene, we created a 5x5 supercell containing 50 carbon atoms, and introduced an additional 20 Å of vacuum space along the *c* lattice direction. For Au and Cu, a 4x4 supercell of the (111) plane was created using periodic slabs, with a vacuum region exceeding 15 Å between vertically repeated slabs. We also employed a perpendicular dipole correction to enhance energy convergence of the adsorbed systems. The model for Au(111) and

Cu(111) slabs consisted of four atomic layers, where the bottom two layers were frozen to the theoretical equilibrium bulk position of Au and Cu, while the two uppermost layers, namely surface layers, were free to relax during the optimization (see Fig. S1 (a)).

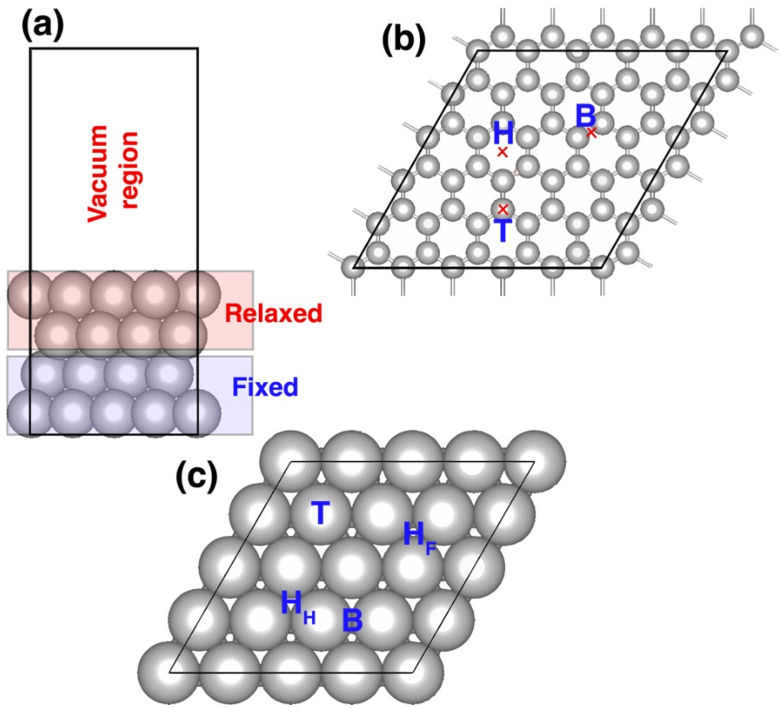

Fig. S1. A schematic illustration of (a) slab model, (b) and (c) the top view of graphene and slab surfaces (Au and Cu) where high symmetric adsorption sites are labeled as T, B, H, H_H, and H_F, representing top, bridge, hollow, hollow-hcp, and hollow-fcc sites, respectively.

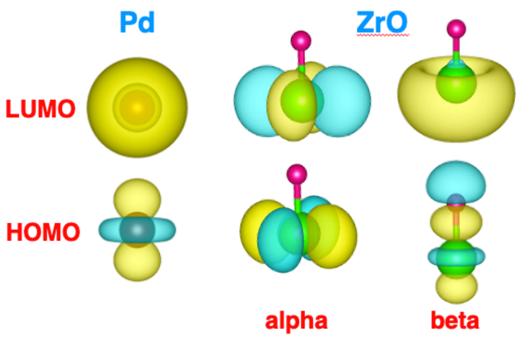

Fig. S2 Molecular orbitals of a single Pd atom and a ZrO superatom.

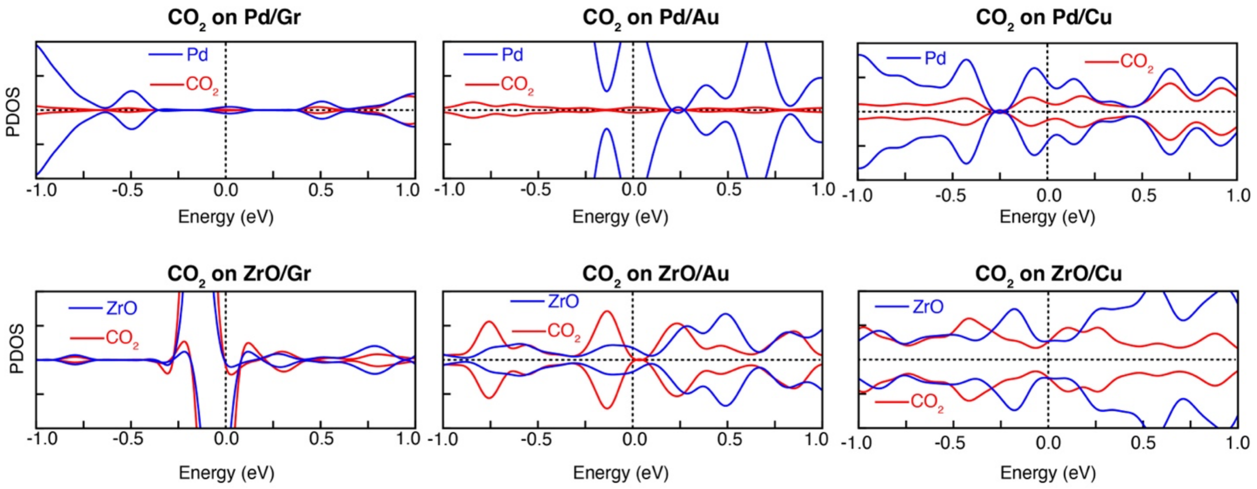

Fig. S3. Partial density of states (PDOS) for the CO_2 molecule's adsorption on Pd (ZrO) supported graphene, Au(111), and Cu(111) where the PDOS of Pd (ZrO) and CO_2 molecule are shown in blue and red colors, respectively. The Fermi energy is set to 0 eV.

Table S2. Total energies (E_{total}), zero-point energies (ZPE), and entropy (S) at T = 298.15 K for $*CO_2$, *COOH, *CO, *CHO, $*CH_2O$, $*CH_2OH$ for the Pd doped Gr, Au, and Cu surfaces.

Pd on Gr	E_{total} (eV)	ZPE (eV)	TS (eV)	G(eV)
$*CO_2$	-489.355	0.309	0.230	-489.276
*COOH	-492.253	0.593	0.264	-491.923
*CO	-482.683	0.194	0.170	-482.658
*CHO	-484.888	0.428	0.163	-4.84.623
$*CH_2O$	-488.908	0.750	0.177	-488.335

*CH_2OH	-492.218	1.062	0.192	-491.348
**Pd on Au**	***E*$_{total}$ (eV)**	**ZPE (eV)**	***TS* (eV)**	***G*(eV)**
*CO_2	-255.390	0.313	0.241	-255.317
*COOH	-258.782	0.600	0.256	-258.438
*CO	-248.588	0.192	0.174	-248.570
*CHO	-251.428	0.438	0.097	-251.087
*CH_2O	-255.063	0.751	0.169	-254.481
*CH_2OH	-258.849	1.078	0.188	-257.959
**Pd on Cu**	***E*$_{total}$ (eV)**	**ZPE (eV)**	***TS* (eV)**	***G*(eV)**
*CO_2	-274.432	0.302	0.214	-274.344
*COOH	-277.891	0.601	0.212	-277.502
*CO	-267.397	0.176	0.065	-267.287
*CHO	-270.261	0.437	0.156	-269.980
*CH_2O	-274.067	0.749	0.168	-273.485
*CH_2OH	-277.720	1.077	0.188	-276.831

Table S3. Total energies (E_{total}), zero-point energies (ZPE), and entropy (*S*) at $T = 298.15$ K for *CO_2, *COOH, *CO, *CHO, *CH_2O, *CH_2OH for the ZrO doped Gr, Au, and Cu surfaces.

**ZrO on Gr**	***E*$_{total}$ (eV)**	**ZPE (eV)**	***TS* (eV)**	***G*(eV)**
*CO_2	-501.650	0.293	0.205	-501.563
*COOH	-505.203	0.612	0.230	-504.820
*CO	-493.498	0.186	0.168	-493.480
*CHO	-497.460	0.462	0.172	-497.170
*CH_2O	-501.808	0.757	0.171	-501.222
*CH_2OH	-505.294	1.082	0.189	-504.400

**ZrO on Au**	$\boldsymbol{E_{total}}$ **(eV)**	**ZPE (eV)**	***TS*** **(eV)**	***G*****(eV)**
$*CO_2$	-268.570	0.305	0.193	-268.458
*COOH	-272.155	0.620	0.199	-271.733
*CO	-260.351	0.183	0.093	-260.261
*CHO	-264.438	0.474	0.143	-264.107
$*CH_2O$	-268.285	0.791	0.141	-267.635
$*CH_2OH$	-271.404	1.103	0.171	-270.472
**ZrO on Cu**	$\boldsymbol{E_{total}}$ **(eV)**	**ZPE (eV)**	***TS*** **(eV)**	***G*****(eV)**
$*CO_2$	-286.930	0.293	0.203	-286.839
*COOH	-290.598	0.623	0.205	-290.180
*CO	-279.062	0.179	0.092	-278.975
*CHO	-282.803	0.454	0.161	-282.509
$*CH_2O$	-287.305	0.772	0.152	-286.685
$*CH_2OH$	-290.482	1.081	0.191	-289.591
